# Carcinome sarcomatoïde: prise en charge thérapeutique et profil pronostique à propos d´une localisation maxillaire inhabituelle (à propos d´un cas)

**DOI:** 10.11604/pamj.2021.38.212.26602

**Published:** 2021-02-24

**Authors:** Zahra Sayad, Bouchra Dani, Rajaa El Azzouzi, Hafsa Elouazzani, Nadia Cherradi, Salma Benazzou, Malik Boulaadas

**Affiliations:** 1 Service de Chirurgie Maxillo-faciale et Stomatologie, Centre Hospitalier Universitaire, Rabat, Maroc,; 2 Laboratoire d´Anatomie Pathologique, Centre Hospitalier Universitaire, Rabat, Maroc

**Keywords:** Carcinosarcome, maxillaire, evidement ganglionnaire, pronostic, à propos d´un cas, Carcinosarcoma, maxillary, node dissection, prognosis, case report

## Abstract

Le carcinome sarcomatoïde est une tumeur maligne rare, agressive de mauvais pronostic à pouvoir récidivant très élevé. Sa localisation au niveau du sinus maxillaire est extrêmement rare. Nous rapportons le cas d´une femme de 42 ans, qui présentait un processus du maxillaire gauche dont une biopsie faite revenant en faveur d´un carcinome sarcomatoïde agressif à métastase ganglionnaire. La patiente a bénéficié d´une exérèse chirurgicale, un évidement ganglionnaire suivi d´une radiothérapie avec une bonne évolution. La rareté de cette tumeur en particulier en cette localisation pose une problématique diagnostique et de prise en charge à temps; cette dernière qui reste à ce jour la controversée. Cependant, une exérèse chirurgicale large reste le meilleur traitement. A travers ce cas nous avons mis le point sur les particularités anatomo-cliniques et surtout pronostiques de cette tumeur.

## Abstract (en)

Sarcomatoid carcinoma is a rare, aggressive, malignant tumor with a poor prognosis and a very high frequency of recurrence. Carcinoma of the maxillary sinus is extremely rare. We report the case of a 42-year-old woman with left maxillary process. Biopsy revealed aggressive sarcomatoid carcinoma with a lymph-node metastasis. The patient underwent surgical excision, lymph-node dissection followed by radiotherapy with good outcome. The rarity of this site-specific cancer poses a problem of diagnosis and timely management which is still a controversial topic. However, wide surgical excision is the gold standard treatment. This study highlights the anatomoclinical peculiarities and, in particular, the prognostic features of this tumor.

## Introduction

Le carcinosarcome est une tumeur maligne biphasique comprenant à la fois une composante carcinomateuse épithéliale et mésenchymateuse. Il s´agit d´une tumeur rare, notamment dans la région tête et cou. Son diagnostic de certitude est histologique et immunohistochimique, qui est souvent tardif ce qui complique sa prise en charge. En raison, du peu de cas rapportés dans la littérature, il n´existe pas de normes standardisées dans le traitement de ce type de cancers. C´est une tumeur de mauvais pronostic, connue par ses récidives aussi bien locales qu´à distance avec un taux de mortalité relativement élevé. Son traitement de choix reste la chirurgie [1, 2].

## Patient et observation

Il s´agit d´une patiente de 42 ans, hypertendue sous traitement avec une mauvaise observance thérapeutique, ayant consulté pour une tuméfaction jugale gauche évoluant depuis 3 ans. L´examen physique trouvait une masse de consistance dure faisant corps à l´os maxillaire gauche, douloureuse, mobile par rapport à la peau, sans signes inflammatoires en regard, avec une hypoesthésie du territoire V2 gauche. En endo buccal on a trouvé une voussure s´étendant depuis la face mésiale de la 24 jusqu´à la 28, qui comble le vestibule et s´étend au palais sans dépasser la ligne médiane avec une muqueuse d´aspect ulcéro-bourgeonnant par endroit. L´examen cervical trouvait une adénopathie submandibulaire gauche mobile et indolore faisant 1,5cm de grand axe.

**Figure 1:**
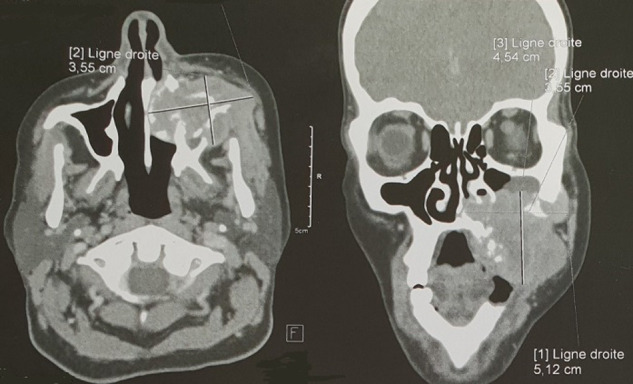
TDM cranio faciale C-C+ en coupes axiales et coronales, fenêtres osseuses et parenchymateuses montrant un processus de densité tissulaire (mesurant 52mmx35mmx45mm) siège au niveau du maxillaire supérieur gauche avec une lyse des parois interne, externe et antérieure du sinus; il s´étend à la fosse nasale homolatérale

La tomodensitométrie (TDM) a mis en évidence un processus tumoral agressif du maxillaire supérieur gauche de densité tissulaire, mesurant 52mm / 31mm, rehaussée de façon hétérogène après injection du produit de contraste, qui s´entend à la fosse nasale homolatérale qu´elle comble arrivant au contact du septum nasal, avec une érosion des parois du sinus antérieure, interne et externe, ainsi qu´une lyse du palais dur gauche (Figure 1). Une biopsie faite qui est revenue en faveur d´un carcinome sarcomatoïde maxillaire. Conformément aux recommandations internationales, la patiente a bénéficié d´une maxillectomie totale par la voie Weber-Fergusson avec curage ganglionnaire homolatéral fonctionnel, suivi d´une radiothérapie à raison de 70 Gray en 35 fractions. Les marges d´exérèse étaient saines. Les suites opératoires étaient simples. Sur un recul de deux ans aucune récidive n´a été décelée.

**Figure 2:**
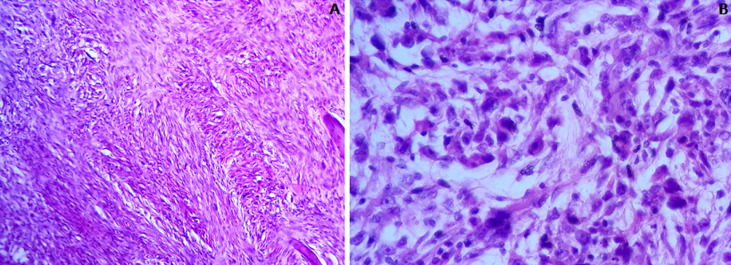
carcinome sarcomatoïde maxillaire: histologiquement, il s´agit d´une prolifération fusocellulaire; A) hématoxyline éosine au Gx10; les atypies cytonucléaires sont marquées avec un pléomorphisme nucléaire; B) hématoxyline éosine au x40

**Figure 3:**
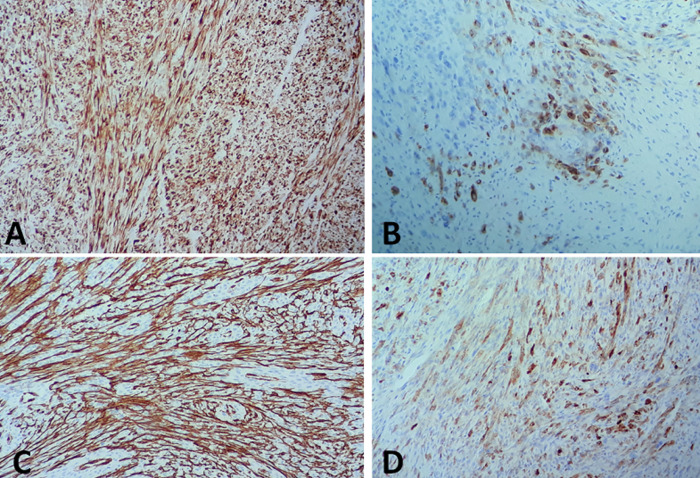
carcinome sarcomatoïde maxillaire: A) à l´étude immunohistochimique les cellules tumorales expriment les marqueurs épithéliaux à savoir la CKAE1/AE3; B) l´EMA; C) les marqueurs mésenchymateux à savoir l´AML; D) la PS100

L´examen anatomopathologique de la pièce d´exérèse opératoire a objectivé macroscopiquement une tranche de section tumorale blanchâtre mal limite d´aspect fasciculé mesurant 7cm de grand axe. Histologiquement, la tumeur répondant à une prolifération tumorale maligne de densité cellulaire élevée, pléomorphe d´architecture fusiforme montrant des atypies cytonucléaires marquées. Les figures de mitoses étaient nombreuses ainsi que les foyers de nécrose tumorale (Figure 2). A l´étude immunohistochimique, les cellules tumorales exprimaient franchement et diffusément CKAE1/AE3 et l´EMA et également l´AML et la PS100. La Désmine, la myogénine, le CD 31 et le CD 34 étaient négatifs (Figure 2, Figure 3). Les marges d´exérèse étaient saines. Les suites opératoires étaient simples. Sur un recul de deux ans aucune récidive n´a été décelée.

## Discussion

Le carcinome sarcomatoïde est une tumeur hautement maligne caractérisée par une double différenciation histologique avec deux composantes: épithéliale et mésenchymateuse ayant un stroma sarcomatoïde. Ces tumeurs constituent moins de 1% de tous les carcinomes de la tête et le cou [3, 4]. Elle touche principalement les patients entre la 5^e^ et 7^e^ décennies avec une prédominance masculine. Cependant, notre patiente est la femme la plus jeune parmi ceux rapportés dans la littérature. Concernant les facteurs de risque, le tabac et l´alcool, l´irradiation, ce qui rejoint le cas de la plupart des autres tumeurs malignes. Chez notre patiente on a noté la notion de tabagisme passif [1, 2].

Aucune symptomatologie clinique n´est spécifique ce qui retarde le diagnostic précoce et complique la prise en charge et augmente la probabilité d´avoir un mauvais pronostic [5]. Au niveau de la tête et du cou, le site de prédilection de cette tumeur est la glande parotide. Tandis que, sa localisation naso-sinusienne est extrêmement rare. A notre connaissance, seulement 19 cas ont été rapportés dans la littérature. En raison de cette rareté, la littérature est pauvre en matière des éléments anatomocliniques et pronostiques, ainsi que prise en charge qui reste encore controversée [6, 7]. L´étiopathogénie évoque plusieurs théories, la plus retenue est la théorie monoclonale qui suggère le potentiel multipotent de la cellule tumorale et son pouvoir de différenciation épithélial et mésenchymateux [1].

En effet, cette différenciation mésenchymateuse, sarcomatoïde est parfois prédominante comme le cas de notre patiente posant le problème de diagnostic différentiel avec un sarcome primitif notamment que des contingents hétérologues de type léiomyosarcome, rhabdomyosarcome ou ostéosarcome peuvent s´observer également dans un carcinome sarcomatoïde d´où la nécessité d´une bonne analyse morphologique et immunohistochimique pour assoir ce diagnostic en mettant en évidence l´expression positive des marqueurs épithéliaux par les cellules tumorales [1, 2, 8]. L´atteinte ganglionnaire cervicale dans ces types de cancers localisés au niveau de la tête et du cou varie entre 7,5% et 26%. C´est le cas de notre patiente, dont la plus grosse adénopathie mesurant 1,5 cm est revenue métastatique à l´examen extemporané d´où on a complété notre geste par un curage ganglionnaire [2].

Il s´agit d´une tumeur très agressive et à caractère infiltrant avec une tendance à la récidive et à métastaser de façon très importante. Cependant, aucune conduite à tenir thérapeutique n´est consensuelle [1, 7, 9, 10]. D´après la littérature, son traitement consiste en une exérèse chirurgicale carcinologique large avec une radiothérapie adjuvante et/ou une chimiothérapie. Dans notre cas, on a réalisé une maxillectomie totale avec un évidement ganglionnaire homolatéral suivi d´une radiothérapie [6, 9]. Le pronostic est lié à la localisation de la tumeur, sa taille, son extension et son stade. L´atteinte naso-sinusienne se présente sous une forme agressive, infiltrante et connue par son caractère récidivant contrairement à d´autres atteintes notamment le larynx et le pharynx. Une chirurgie première avec une radiothérapie adjuvante permet d´améliorer le pronostic, de réduire le taux de récidives locales ainsi que le taux de mortalité global de cette maladie. Le taux de survie à 5 ans varie de 40% à 60% en fonction du stade de la tumeur [1, 9, 10].

## Conclusion

Le carcinosarcome maxillaire est une entité rare et agressive avec un mauvais pronostic en rapport avec son pouvoir récidivant important aussi bien locale qu´à distance. Sa prise en charge thérapeutique est base sur la chirurgie avec une radiothérapie adjuvante voir une chimiothérapie. On tient a souligné à travers cette observation l´importance d´expliquer au patient le caractère agressif de la tumeur, son mauvais pronostic et la possibilité de récidive et le suivi à long terme même après un traitement bien conduit.

## References

[1] Sepúlveda I, Frelinghuysen M, Garcia C, Spencer ML, Platin E, Alarcon J (2014). Maxillary Carcinosarcoma: A case report and review of the literature. An International Journal.

[2] Altınay S, Altinok A, Süt PA, Taskın U, Bilici A (2018). Spindle cell carcinoma (sarcomatoid carcinoma) of maxillary sinus and nasal cavity with orbital involvement: a rare case report and brief review of literature. Dent Oral Craniofac Res.

[3] Hasnaoui J, Anajar S, Tatari M, Abada R, Rouadi S, Roubal M (2017). Carcinosarcoma of the maxillary sinus: a rare case report. Ann Med Surg (Lond).

[4] Thompson LD, Wieneke JA, Miettinen M, Heffner DK (2002). Spindle cell (sarcomatoid) carcinomas of the larynx: a clinicopathologic study of 187 cases. Am J Surg Pathol.

[5] Moon JK, Kim AY, Chang DS, Park KY (2013). Carcinosarcoma of the maxillary sinus. Clin Exp Otorhinolaryngol.

[6] Alem HB, AlNoury MK (2014). Management of spindle cell carcinoma of the maxillary sinus. Am J Case Rep.

[7] Guan M, Li Y, Shi ZG, Xie LS, Cao XL (2014). Sarcomatoid carcinoma involving the nasal cavity and paranasal sinus: a rare and highly progressive tumor. Int J Clin Exp Pathol.

[8] Kumar M, Goyal S, Bahl A, Das P, Sharma DN, Ray R (2008). Sarcomatoid carcinoma of the maxillary sinus: a rare head and neck tumor. J Cancer Res Ther.

[9] Patel TD, Vazquez A, Plitt MA, Baredes S, Eloy JA (2015). A case econtrol analysis of survival outcomes in sinonasal carcinosarcoma. Am J Otolaryngol.

[10] Cheong JP, Rahayu S, Halim A, Khir A, Noorafidah D (2014). Report of a rare case of carcinosarcoma of the maxillary sinus with sternal metastasis. Ear Nose Throat J.

